# Resistance Training With Partial Blood Flow Restriction in a 99-Year-Old Individual: A Case Report

**DOI:** 10.3389/fspor.2021.671764

**Published:** 2021-06-22

**Authors:** Maíra Camargo Scarpelli, João Guilherme Almeida Bergamasco, Estevan A. de Barros Arruda, Summer B. Cook, Cleiton Augusto Libardi

**Affiliations:** ^1^MUSCULAB – Laboratory of Neuromuscular Adaptations to Resistance Training, Department of Physical Education, Federal University of São Carlos, São Carlos, Brazil; ^2^Department of Kinesiology, University of New Hampshire, Durham, NH, United States

**Keywords:** strength training, sarcopenia, older adults, nonagenarians/centenarians, vascular occlusion

## Abstract

In aging populations for which the use of high loads is contraindicated, low load resistance training associated with blood flow restriction (RT-BFR) is an alternative strategy to induce muscle mass gains. This study investigates the effects of RT-BFR on muscle mass, muscle function, and quality of life of a 99-year-old patient with knee osteoarthritis and advanced muscle mass deterioration. Training protocol consisted of 24 sessions of a unilateral free-weight knee extension exercise associated with partial blood flow restriction through a manometer cuff set at 50% of complete vascular occlusion pressure. We evaluated: cross-sectional area (CSA) and thickness (MT) of the vastus lateralis muscle by ultrasound; function through the Timed Up and Go (TUG) test; and quality of life (QoL) by the WHOQOL-bref, WHOQOL-OLD and WOMAC questionnaires. All tests were performed prior to the training period (Pre) and after the 12th (Mid) and 24th (Post) sessions. Changes were considered significant if higher than 2 times the measurement's coefficient of variation (CV). After 24 sessions, there was an increase of 12% in CSA and 8% in MT. Questionnaires scores and TUG values worsened from Pre to Mid and returned in Post. We consider RT-BFR a viable and effective strategy to promote muscle mass gains in nonagenarians and delay the decline in functionality and QoL associated with aging.

## Introduction

The aging process is associated with a gradual general decline in physical performance, functionality and its underlying biological mechanisms (Mitchell et al., 2012; Billot et al., 2020). A critical aspect of this decline is the decrement in muscle quantity and quality, which is characterized by a decrease in the cross sectional area of muscle fibers (Narici et al., 2003) and an increase in non-contractile components intertwined with muscle tissue (e.g., adipose and connective tissue) (Visser et al., 2002). Some studies indicate that the rate of muscle loss accelerates at more advanced ages (e.g., 80+ years old) (Baumgartner et al., 1995; Janssen et al., 2000; Kyle et al., 2001), further compromising the ability to independently perform activities of daily living (Aagaard et al., 2010; Cruz-Jentoft et al., 2010). The loss of muscle mass can also contribute to the development of other pathologies associated with aging, such as osteoarthritis (OA) (Shorter et al., 2019), a progressive musculoskeletal disease commonly accountable for debilitating pain and loss of function and quality of life (Glyn-Jones et al., 2015).

Resistance training (RT) performed with high loads (RT-HL) has historically been prescribed as the gold standard for the optimization of morphological and functional skeletal muscle adaptations (Fiatarone et al., 1994; ACSM, 2009), and thus as an effective and safe approach to attenuate the effects of aging regardless of age (Grgic et al., 2020). However, using high loads may imply an intolerably high mechanical overload and a contraindicated burden for populations of older ages who have been enduring the gradual decline in muscle mass for long, especially if they suffer from OA and present some joint limitation (Loenneke et al., 2011; Hughes et al., 2017). In this sense, low load RT associated with blood flow restriction (RT-BFR) has been presented as an alternative, as it promotes gains in strength and muscle mass similar to RT-HL with a reduced mechanical overload (Laurentino et al., 2008; Yasuda et al., 2014; Libardi et al., 2015; Lixandrao et al., 2018; Patterson et al., 2019).

Still, evidence of the effects of RT-BFR in adults aged 90 or older is scarce. In fact, to best of our knowledge, there are no reports of RT-BFR being applied in nonagenarians or older in the current literature. Thus, the present case study aimed at adding to the body of evidence by evaluating the effects of RT-BFR on muscle mass, mobility, and quality of life in a 99-year-old patient with knee osteoarthritis and advanced muscle mass deterioration, which caused pain and affected his performance in activities of daily living, balance, and gait.

## Case Description

### Participant's Characteristics

The study reports the case of a 99-year-old man (body mass = 55 kg; height = 160 cm; thigh circumference: right = 34 cm, left = 35 cm), with severe muscle mass deterioration and knee osteoarthritis, accompanied by muscle and joint pain that affected his muscle strength and function. Despite encountering great difficulty, he was able to walk, sit down and stand up with only the assistance of a walking stick; other simple activities of daily living, such as bathing and dressing, required the help of a family member. Following medical guidance, he made continuous use of medications for muscle-joint pain (Ultracet: tramadol hydrochloride 37.5 mg, paracetamol 325 mg; Tandrilax: caffeine 30 mg, carisoprodol 125 mg, diclofenac sodium 50 mg, paracetamol 300 mg) and anxiety (Bromazepan 3 mg). In addition to pharmacological treatment, he performed 3 weekly physical therapy sessions to relieve pain and reduce joint stiffness. He did not present any other acute or chronic diseases. The study was approved by the Institution's ethics committee and the participant and family members were informed of all benefits and risks of the study before signing an institutionally approved informed consent form.

### Data Collection

**Figure 1:**
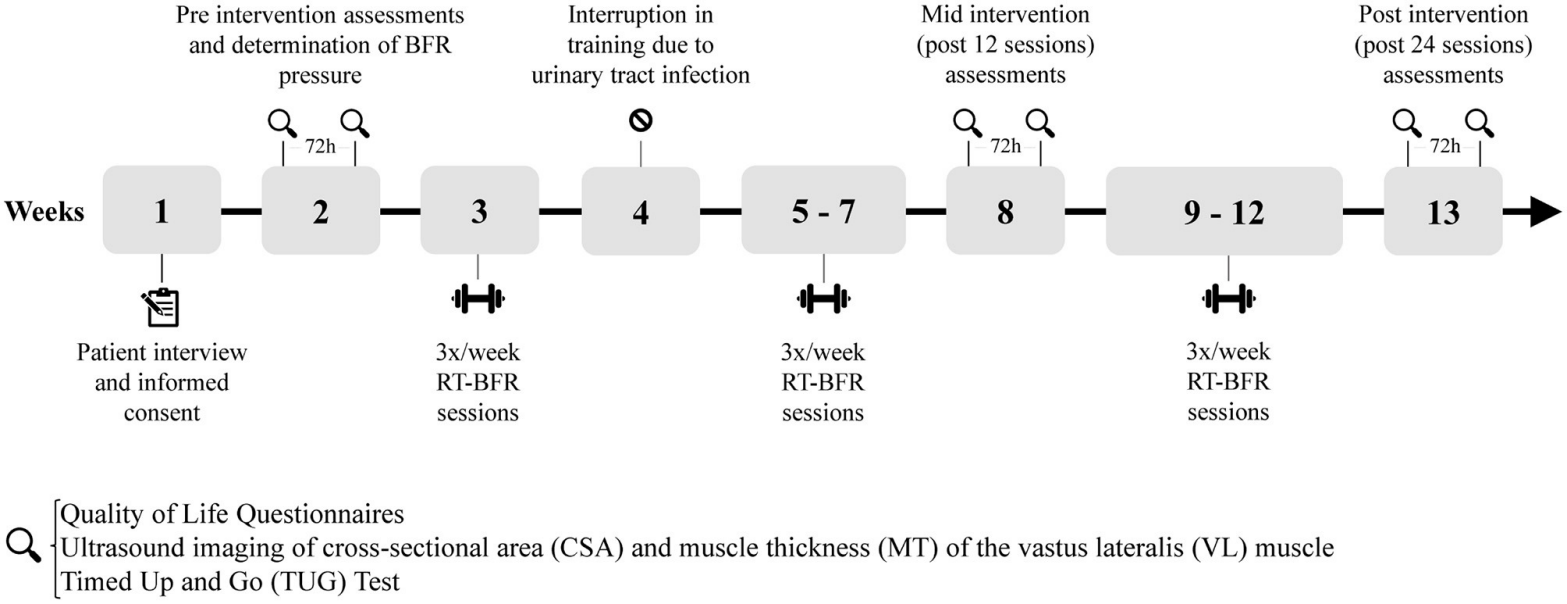
Timeline of intervention.

We assessed the cross-sectional area (CSA) and muscle thickness (MT) of the vastus lateralis (VL) muscle, functional performance and quality of life {The World Health Organization Quality of Life Questionnaires [WHOQOL-bref (The WHOQOL Group, 1998) and WHOQOL-OLD (Power et al., 2005)] and The Western Ontario and McMaster Universities Questionnaire (Bellamy et al., 1988) [WOMAC]}, before (Pre) the training period and after 12 (Mid) and 24 (Post) training sessions (Figure 1). At each of these time points, 2 assessments were performed on two different days at the same time of day and the average between the two was presented. The participant was instructed to maintain his eating routine and abstain from stressful activities for at least 72 h before each evaluation day (Newton et al., 2008; Damas et al., 2016).

**Figure 2:**
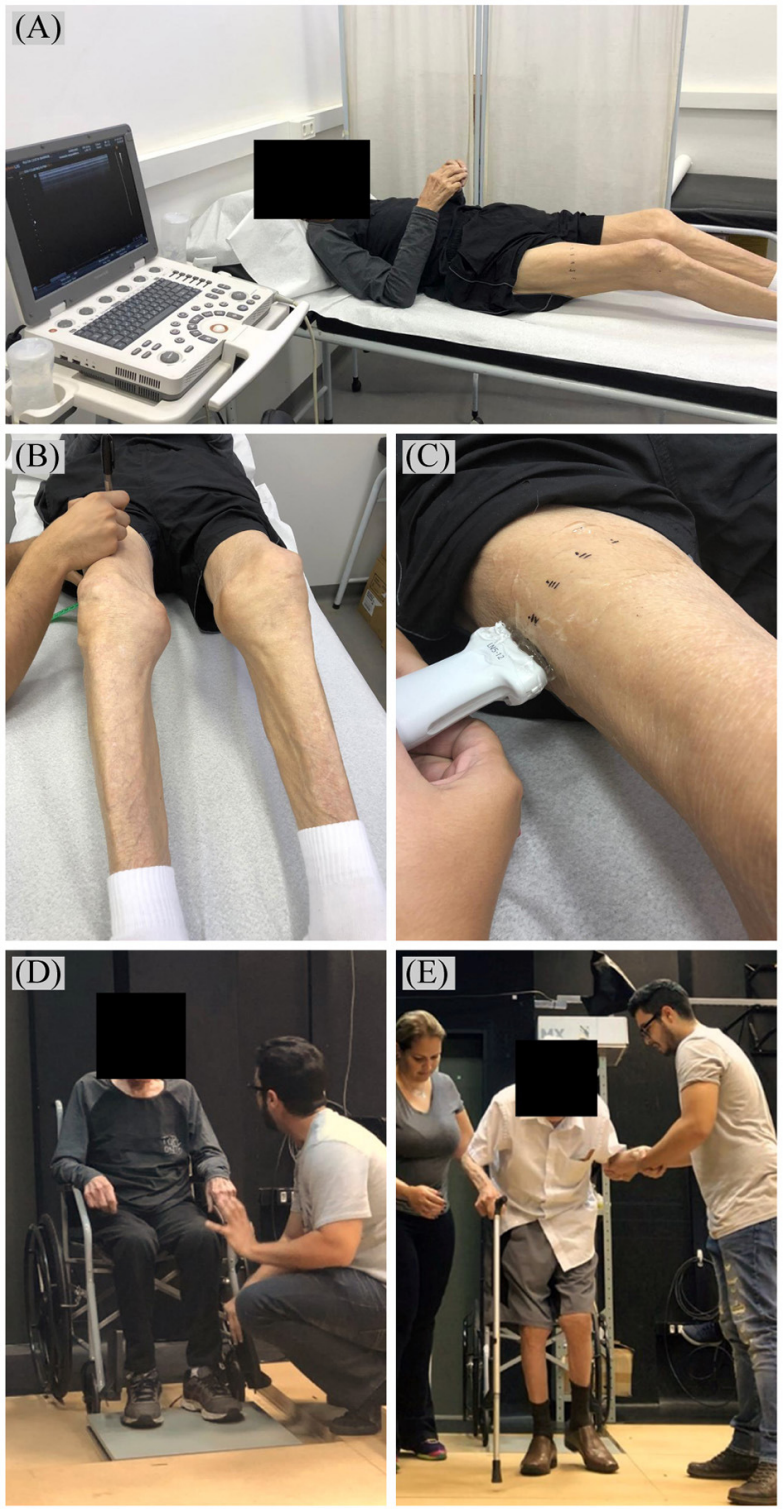
**(A)** Positioning and **(B)** skin markings for ultrasound image acquisition of vastus lateralis cross-sectional area and **(C)** muscle thickness (with ultrasound probe oriented longitudinally to the muscular belly). Timed Up and Go Test **(D)** instructions and **(E)** execution.

The CSA of the VL was evaluated using an ultrasound device (US) (Lixandrao et al., 2014). Images were acquired using mode B ultrasound, with a linear probe configured at 7.5 MHz (Samsung, MySono U6, Brazil), and applying transmission gel to ensure acoustic coupling with minimal epidermal compression. The skin was marked at the point corresponding to 50% of the total length of the right femur (SENIAM, 2005); from this point, successive markings were made transversely, both medially and laterally, at 2 cm intervals, to guide the displacement of the probe in the sagittal plane (Figure 2). Sequential images of the right VL were recorded every 2 cm, starting at the most medial skin mark (over the rectus femoris muscle), and moving in the mid-lateral direction. An investigator, blinded to the timepoints, then opened the captured images in PowerPoint (Microsoft, USA), rotated them manually and organized them to reconstruct the complete view of the cross section of the VL. The complete image was then opened on the Image J software and the “polygonal” tool was used to calculate the CSA. MT was evaluated at the same time and place of acquisition of the CSA images, but with the probe oriented longitudinally to the muscular belly. The “straight” tool of the Image J software (Erskine et al., 2009) was used to measure the vertical distance between the deep aponeurosis and the superficial muscle aponeurosis at the right and left margins of the image and the mean value was recorded. Two US images were evaluated following this same procedure and the average between them was presented as the final MT value. The CV of the CSA and MT measures were 4.25 and 2.96%, respectively.

Following US imaging, a personalized blood pressure cuff (175 × 94 mm) with a pressure gauge (Missouri, São Paulo, Brazil) was fixed in the proximal region of his right thigh and a vascular doppler probe (DV-600; Martec, Ribeirão Preto, Brazil) was placed over his tibial artery. For the determination of blood pressure of complete vascular occlusion at rest, the cuff was inflated until the moment when the auscultatory pulse of the tibial artery was interrupted (Libardi et al., 2015). The procedure was repeated for the left leg. The cuff pressure necessary for complete blood flow restriction was 170 mmHg for the right leg and 150 mmHg for the left leg. Cuff pressure to be used during exercise was established at 50% of these values.

The functional performance was measured through “Timed Up and Go” (TUG) test with the aid of a force platform (AccuGait, AMTI, USA). The participant's wheelchair was positioned–and secured–so that his feet were placed on the platform (Figure 2). He started the test sitting with his back on the chair, arms positioned on the side arms and feet on the force platform until the start command. After the command, the participant was instructed to get up as quickly as possible using the armrests of the chair, then walk–with external assistance–a distance of 3 m, return, and sit again with the back against the seat (Miotto et al., 1999). The first trial was a familiarization session and the second trial always served as the test session. Balance Clinic software (AMTI, USA) was used to compute the total time to complete the task. The TUG time was also divided into two components: (1) time spent atop the force plate at the beginning and ending of the test, which can approximately reflect the time used to get up and sit back on the chair, and (2) time spent off the force plate, i.e., time in which no forces are being applied to the platform, and thus, can approximately reflect the walking portion of the test. The CV's for the full TUG test, the time spent on the platform and time spent off the platform were of 5.98, 6.86, and 7.26%, respectively.

Three questionnaires were applied to assess possible impacts of the intervention on the participants quality of life (QoL): WHOQOL-bref, WHOQOL-OLD and WOMAC. The first one is a short form QoL assessment composed by one general QoL item, one general health item, and 24 items that cover four specific domains: physical health, psychological, social relationships and environment (The WHOQOL Group, 1998). The WHOQOL-OLD is a supplementary module which contains six facets considered especially relevant to older adults: (1) “sensory abilities” (2) “autonomy,” which refers to the ability to live independently and make his own decisions; (3) “past, present, and future activities,” related to accomplishments in life; (4) “social participation”; (5) “death and dying,” related to concerns and fear of dying; and, (6) “Intimacy” (Power et al., 2005). The WOMAC questionnaire is composed of three dimensions (pain, stiffness, and physical function) and is used to assess clinically relevant changes in health related QoL as a result of an intervention for people with OA (Bellamy et al., 1988). Scores for WHOQOL-bref and WHOQOL-OLD are scaled from 0 to 100 in a positive direction (i.e., higher scores denote higher QoL) while scores for WOMAC are scaled from 0 to 100 in a negative direction. The CV's for the global scores of WHOQOL-bref, WHOQOL-OLD, and WOMAC were of 2.97, 9.00, and 3.36%, respectively.

### Resistance Training Associated With Blood Flow Restriction

**Figure 3:**
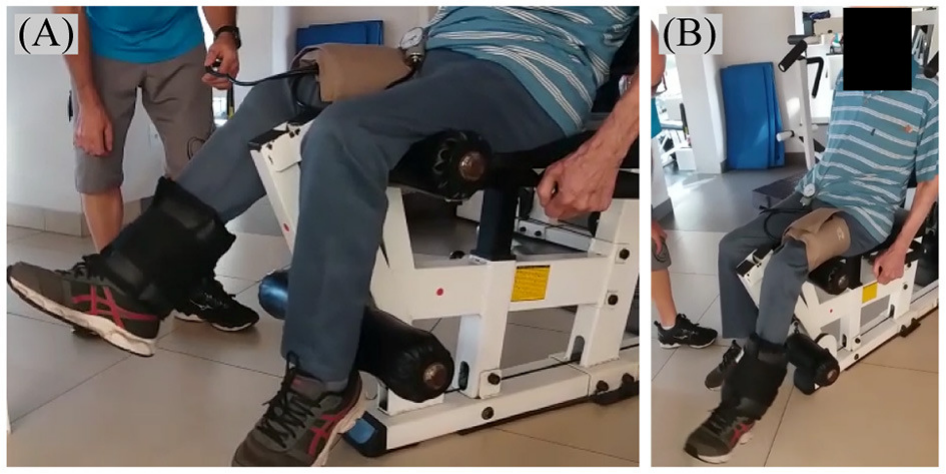
**(A,B)** Unilateral free-weight knee extension exercise with blood flow partially restricted by a blood pressure cuff (175 × 94 mm) fixed in the proximal region of the thigh.

Training sessions were held three times per week, for a total of 24 sessions. The RT-BFR consisted of a unilateral free-weight knee extension exercise (Figure 3). The participant was instructed to sit upright on a knee extension chair, with his lower back supported by the chair back, and then was instructed to perform the exercise with the largest range of motion possible to achieve without pain. He started the protocol with a warm-up of 10 repetitions without overload; then he performed three sets of 10–15 maximum repetitions using ankle weights strapped around his shin with a blood flow restriction (described below). A 1-min interval was allowed between sets. If the participant performed less than 10 repetitions in the first set, the load was readjusted to achieve at least 10 repetitions in the next; if 15 repetitions (or more) were performed in each of the three sets, the load adjustment was performed in the next session. In the first session of training, only the limb weight was used; by the last session, load was progressed to a 4 kg ankle weight. After performing the training with one leg, it was repeated with the other. The starting leg was switched every session. Blood flow was partially restricted unilaterally during sets and intervals using a cuff (175 × 94 mm; Missouri, Brazil) with a pressure gauge positioned in the proximal region of the thigh. Training pressure was established at 50% of the pressure of complete vascular occlusion (right leg: 85 mmHg; left leg: 75 mmHg) and was readjusted throughout the protocol so that the relative occlusion was maintained (Patterson et al., 2019).

### Data Analysis

From the tests (T), a coefficient of variation (CV) was computed for each outcome as:

(1) $$\begin{array}{r} CV=\;\left[\frac{TE}{{\bar{T}}_{ij}}\right]\times 100 \end{array}$$

(2) $$\begin{array}{r} TE=\;\frac{SD\left(T_{i2}-\;T_{i1}\right)}{\sqrt{2}} \end{array}$$

(3) $$\begin{array}{r} {\bar{T}}_{ij}=\frac{\sum_{i}\sum_{j}\left(T_{ij}\right)}{\left(i\times j\right)} \end{array}$$

where TE is the typical error of measurement and SD is the standard deviation of the difference between the two tests T (*j* = 1, 2) made on time point *i* (*i* = Pre; Mid; Post). Thus, the CV is an alternative form of presenting the TE, expressed as a percentage of the subject's mean score (Hopkins, 2000). It has been suggested that 1.5–2 times the typical error (TE) of measurement can be used as a threshold for deciding if a real change in outcome has occurred (Hopkins, 2000). Therefore, we evaluated percentage changes and compare with two times the CV as the cut point for significance.

## Results

Results from the Pre, Mid, and Post intervention assessments, percentage changes and the coefficients of variation are depicted in Table 1. The participant presented a significant increase of 12.11% in CSA and of 8.37% in MT. There was a non-significant reduction of 1.7% in the performance of the TUG mobility test. The questionnaires resulted in scores that, in general, behaved similarly across the intervention period: after 12 training sessions there was a worsening in his perception of QoL but after 24 sessions scores returned to initial values. None of the global scores presented significant changes from Pre to Post intervention. We can anecdotally report that the participant reported slight discomfort from the pressure of the cuff, but no pain during or after training sessions, apart from regular post-exercise soreness following the first 3–4 sessions. No adverse events related to the intervention were reported by either the participant or his family members.

**Table 1:** Vastus lateralis cross-sectional area and thickness, functionality (timed up and go) and quality of life scores before the intervention (Pre), after 12 (Mid), and 24 (Post) sessions of resistance training associated with partial blood flow restriction.

	**Pre**	**Mid**	**Post**	**Pre to Mid (%)**	**Mid to Post (%)**	**Pre to Post (%)**	**2xCV (%)**
**Muscle cross-sectional area (cm**^**2**^**)**	2.91	3.03	3.27	3.83	7.98	12.11^*^	8.50
**Muscle Thickness (cm)**	0.92	0.90	1.00	−2.44	11.08^*^	8.37^*^	5.92
**TUG (s)**	107.34	123.48	105.52	15.04^*^	−14.54^*^	−1.70	11.96
Time spent out of the force platform	68.88	86.12	72.32	25.03^*^	−16.02^*^	4.99	14.51
Time spent on the force platform	38.46	37.36	33.2	−2.86	−11.13	−13.68	13.72
**WHOQOL-bref^#^**	52.08	36.43	51.34	−30.05^*^	40.91^*^	−1.43	5.94
General quality of life	50.00	25.00	50.00	−50.00^*^	100.00^*^	0.00	0.00
General health	50.00	25.00	50.00	−50.00^*^	100.00^*^	0.00	0.00
Physical health	50.00	32.14	39.29	−35.71^*^	22.22	−21.43	28.82
Psychological	58.33	41.67	54.17	−28.57^*^	30.00^*^	−7.14^*^	0.00
Social relationships	41.67	41.67	58.33	0.00	40.00^*^	40.00^*^	0.00
Environment	62.50	53.13	56.25	−15.00^*^	5.88^*^	−10.00^*^	0.00
**WHOQOL-OLD^#^**	52.08	45.83	52.08	−12.00	13.64	0.00	18.00
Sensory abilities	50.00	43.75	43.75	−12.50	0.00	−12.50	44.54
Autonomy	50.00	37.50	56.25	−25.00^*^	50.00^*^	12.50^*^	0.00
Past, present, and future activities	50.00	43.75	56.25	−12.50^*^	28.57^*^	12.50^*^	0.00
Social participation	56.25	62.50	56.25	11.11^*^	−10.00^*^	0.00	0.00
Death and dying	50.00	50.00	50.00	0.00	0.00	0.00	81.65
Intimacy	56.25	37.50	50.00	−33.33^*^	33.33^*^	−11.11^*^	0.00
**WOMAC^##^**	66.15	77.08	68.23	16.54^*^	−11.49^*^	3.15	6.72
Pain	47.50	55.00	65.00	15.79	18.18	36.84^*^	33.51
Stiffness	62.50	68.75	62.50	10.00	−9.09	0.00	15.80
Physical function	72.06	81.62	70.59	13.27^*^	−13.51^*^	−2.04	5.79

# *WHOQOL-bref and WHOQOL-OLD scores are scaled from 0 to 100 in a positive direction: the maximum value (100) indicates better quality of life and the minimum value (0), worse*.

## *WOMAC scores are scaled from 0 to 100 in a negative direction: the maximum value (100) indicates worse quality of life and the minimum value (0), better*.

* *Percentage changes >2 times the coefficient of variation (CV)*.

## Discussion

This case report suggests that RT-BFR is an effective strategy to promote positive effects on parameters of muscle hypertrophy, functional performance, and quality of life of a nonagenarian with severe muscle loss and osteoarthritis. Previous studies had already demonstrated that RT-BFR was an efficient alternative strategy to promote neuromuscular adaptations in older individuals, including ones with mobility limitations (Fry et al., 2010; Yasuda et al., 2014; Libardi et al., 2015; Vechin et al., 2015; Cook et al., 2017; Hughes et al., 2017; Lixandrao et al., 2018). However, to the best of our knowledge, this is first study to apply this training method and evaluate its effects in a nonagenarian.

Before the commencement of the intervention, our subject presented a right thigh circumference of 34 cm and a VL CSA and MT of 2.91 cm^2^ and 0.92 cm, respectively. A recent study by Cook et al. (2017) evaluated a group of 36 older adults classified as being at risk of mobility limitations before various strength training interventions. They were aged 67–92 years old and had a mean VL CSA of 14.9 ± 4.3 cm^2^. Yet, out of the 36 participants, only two were nonagenarians: a 90-year-old female with a right leg circumference of 54.9 cm and baseline VL CSA of 13.2 cm^2^ and a 92-year-old male with a right leg circumference of 46.3 cm and VL CSA of 11.5 cm^2^. The large difference between baseline values of Cook et al.'s participants and the values presented by our subject suggest he could be in a far more advanced stage of muscle mass loss. This suggestion is corroborated by the fact that Cook et al.'s participants were classified as being at risk of mobility limitations but did not require assistance with walking, such as our subject did. At the end of the training, our subject had an increase of 12.1% in the CSA and 8.4% in the MT of his VL. These gains have proven significant since they surpass 2 times the CV of the VL CSA and MT measurements (i.e., 8.5 and 5.9%, respectively). Comparatively, in the study of Cook et al. (2017), after 12-weeks of training the mean increase in the VL CSA via MRI was of 7.7 ± 4.7% and 7.5 ± 9.2% for the RT-HL and RT-BFR groups, respectively. Yet, the nonagenarian participants presented results very distinct from the group means: the 90-year-old female participated in RT-HL and presented a muscle mass decrease of −0.1% in the VL CSA, while the 92-year-old male was in the control group and presented an increase of 0.1% in the VL CSA. Thus, although starting off from much lower Pre values and despite the ~10-year age difference, our subject still showed significant muscle mass gains. Yet, these gains were not enough to reach absolute values of muscle mass similar to the values of Cook et al.'s participants.

The TUG test is considered an easy-to-use assessment of functional performance. The performance on the test relates to balance, gait and functional capacity of older adults and may indicate their degree of frailty and assist in the diagnosis of sarcopenia (Podsiadlo and Richardson, 1991; Cruz-Jentoft et al., 2010). Older adults who take longer than 13.5 s to complete the test are considered to be at risk for falls, with a 90% prediction rate (Podsiadlo and Richardson, 1991). Breaking up the TUG test in component tasks (standing up from a seated position, walking, turning, stopping, and sitting down) and evaluating the time to complete each of them separately provides useful clinical information which may guide future interventions (Wall et al., 2000). Despite a noticeable qualitative improvement in gait parameters, our subject did not present significant changes on either the time spent atop the force platform, i.e., time used for standing up and sitting back down, or the time related to the walking component of the test. The TUG time was reduced by 1.70%, a non-significant change since it was smaller than 2xCV of 11.96%. Absolute values represent a much lengthier performance than what is presented in the literature. A meta-analysis by Bohannon (2006) indicated that the mean (95% confidence interval [CI]) TUG time for healthy older adults from 80 to 99 years old was 11.3 (10.0–12.7) s. Lusardi et al. (2003) report a TUG time of 23.4 ± 9.2 s (16.6–30.3; 95% CI) for men between 90 and 101 years old. Therefore, values imply our participant had a high degree of frailty which did not improve with training. However, TUG results seem to indicate that the intervention prevented further loss in functionality performance. Cadore et al. (2014) showed that a no-intervention control group (age 90.1 ± 1.1 years) significantly reduced strength and functional outcomes after 12 weeks, despite having maintained muscle CSA in the same period. Thus, it is plausible to suggest that maintenance of TUG scores denotes a clinical significance of the increase in muscle mass observed in our participant.

As for QoL assessments, none of the global scores showed significant variations from pre to post-training. This is in agreement with results from Cook et al. (2017) which showed no differences in QoL following any type of training. In the WHOQOL-bref analysis, the score for the “Social relationships” domain presented a 40% increase from Pre to Post values. This may be partly due to the care and attention from researchers and the time spent with training instructors (Dickens et al., 2011; Franke et al., 2021). Unpublished data from Cook et al. indicates that older adults that have participated in research interventions listed the researchers as people in their social support system. The increase in “Social relationships” was high enough to counterbalance the reductions observed in the “Physical health,” “Psychological” and “Environment” domains. Among the WHOQOL-OLD components, “Autonomy” and “Past, present and future activities” facets scores improved and counterbalanced the decreases in scores for “Sensory abilities” and “Intimacy.” However, there is no consensus as to whether exercise training has indeed a positive effect on QoL in frail older adults (Chou et al., 2012; Campbell et al., 2021). In the WOMAC Index, the “Pain” dimension stands out with a significant increase of ~37%, indicating a worsening in his perception of pain. However, WOMAC instructions ask the patient to rate the items according to the last 72 h, thus making it difficult to distinguish if the score variation was a result from the intervention or could be related to an acute pain episode related to the knee osteoarthritis.

The TUG test as well as the scores on the QoL questionnaires allow us to observe a pattern of behavior, in which results worsen after 12 training sessions and return to their initial values after 24 sessions. A possible explanation for these results lies in the fact that in the second week of the intervention period our subject was diagnosed with a urinary tract infection which demanded training to be interrupted for a week. Family members reported that the infection caused a reduction in the participants level of activity, in his independence for activities of daily living, as well as in his eating habits. For instance, the walking cane was no longer enough as an aid for mobility and the use of a walker was required. When the infectious condition was resolved, training was resumed. It can be argued that the one-week interruption in training was not sufficient to impair muscle mass gains. Evidence from Hakkinen et al. (2000) indicated that a 3-week detraining was not enough to negatively affect muscle mass changes in older subjects. However, this study included subjects aged 62–78 years old, who possibly had a most preserved muscle mass (Janssen et al., 2000; Kyle et al., 2001). The interruption due to the urinary tract infection did deteriorate his apparent liveliness and disposition. Thus, this could still have been reflected in the functionality and QoL evaluations carried out after the first 12 sessions. It could be speculated that the RT-BFR may have possibly accelerated the recovery process, allowing quality of life and functionality parameters to return to the initial values after 24 sessions.

The present study has some limitations. Assessments of QoL refer to a subjective evaluation of multiple components that may be influenced by several other underlying factors not related to the proposed intervention. Yet, the questionnaires applied are widely used and accepted throughout the literature (Skevington et al., 2004; Power et al., 2005; Bellamy et al., 2010). Also, this is an individual report, and the paucity of comparison cases limits inferences on intervention efficacy and outcomes. Further investigation is necessary to allow extrapolation and generalization of conclusions. Still, we expect our findings may encourage future clinical trials to investigate the effects of these methods in this age group. Based on the currently available literature, the observed differences amongst nonagenarians, as well as between their outcomes and the group values observed when only sexagenarians to octogenarian subjects are included in the analysis, make it a challenge to conjecture as to the most effective and safe training interventions for this population. Since the severity of functional decline tends to gradually increase over the years (Valenzuela et al., 2019), the report of studies involving participants aged 90 or older, is extremely relevant.

We conclude that RT-BFR contributed to increase muscle mass and delay the progressive decline of muscle function and quality of life. Our findings suggest that RT-BFR is a viable non-pharmacological strategy that can be applied for this purpose and in this age group, with simple equipment and in the elderly's own home.

## Data Availability Statement

The raw data supporting the conclusions of this article will be made available by the authors, without undue reservation.

## Ethics Statement

The studies involving human participants were reviewed and approved by Research Ethics Committee of the Federal University of São Carlos. The patients/participants provided their written informed consent to participate in this study. Written informed consent was obtained from the individual(s) for the publication of any potentially identifiable images or data included in this article.

## Author Contributions

The study was conceived and designed by MS, JB, EA, and CL. MS, JB, and EA were responsible for the acquisition, analysis, and interpretation of data. MS, SC, and CL were responsible for writing. All authors critically reviewed and approved the final manuscript.

## Conflict of Interest

The authors declare that the research was conducted in the absence of any commercial or financial relationships that could be construed as a potential conflict of interest.
